# Marie and BERT—A Knowledge Graph Embedding Based Question Answering System for Chemistry

**DOI:** 10.1021/acsomega.3c05114

**Published:** 2023-08-25

**Authors:** Xiaochi Zhou, Shaocong Zhang, Mehal Agarwal, Jethro Akroyd, Sebastian Mosbach, Markus Kraft

**Affiliations:** † Department of Chemical Engineering and Biotechnology, University of Cambridge, Philippa Fawcett Drive, Cambridge CB3 0AS, U.K.; ‡ CARES, Cambridge Centre for Advanced Research and Education in Singapore, 1 Create Way, CREATE Tower, #05-05, Singapore 138602, Singapore; § CMCL Innovations, Sheraton House, Castle Park, Cambridge CB3 0AX, U.K.; ∥ School of Chemical and Biomedical Engineering, Nanyang Technological University, 62 Nanyang Drive, Singapore 637459, Singapore; ⊥ The Alan Turing Institute, London NW1 2DB, U.K.

## Abstract

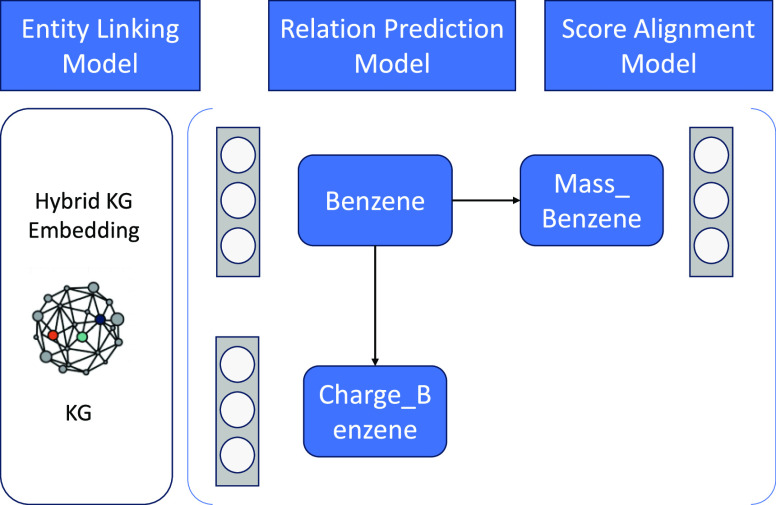

This paper presents a novel knowledge graph question answering (KGQA) system for chemistry, which is implemented on hybrid knowledge graph embeddings, aiming to provide fact-oriented information retrieval for chemistry-related research and industrial applications. Unlike other existing designs, the system operates on multiple embedding spaces, which use various embedding methods and queries the embedding spaces in parallel. With the answers returned from multiple embedding spaces, the system leverages a score alignment model to adjust the answer scores and rerank the answers. Further, the system implements an algorithm to derive implicit multihop relations to handle the complexities of deep ontologies and improve multihop question answering. The system also implements a BERT-based bidirectional entity-linking model to enhance the robustness and accuracy of the entity-linking module. The system uses a joint numerical embedding model to efficiently handle numerical filtering questions. Further, it can invoke semantic agents to perform dynamic calculations autonomously. Finally, the KGQA system handles numerous chemical reaction mechanisms using semantic parsing supported by a Linked Data Fragment server. This paper evaluates the accuracy of each module within the KGQA system with a chemistry question data set.

## 1 Introduction

In the rapidly evolving digital world, the chemistry sector is producing an enormous amount of data, which is becoming increasingly complex to handle. The traditional methods of information storage and retrieval are inadequate to manage this vast quantity of information. In this context, knowledge graphs (KGs) provide a flexible and powerful framework for semantic retrieval, enabling efficient storage and retrieval of complex and interconnected data. KGs can also uncover new relationships between different entities in the data. By conducting a deep exploration of the chemical space using KGs, researchers can effectively navigate the vast and complex chemical space to uncover previously unknown relationships between chemical entities, further expanding their understanding of the chemical space and its potential applications. However, KGs are usually very large and not easily accessible for users as they need to know a query language as well as the structure and relations in the KG.

KG question answering (KGQA)^1–4^ systems bridge human users to the complex knowledge within KGs through natural language queries by providing a natural language interface for querying KGs and accessing relevant information. This allows users to perform more complex and nuanced searches than would be possible by using traditional search methods. Thus, the application of deep exploration of the chemical space using KGQA systems has the potential to revolutionize the information discovery process and pave the way for more efficient and effective utilization of chemical data across a wide range of fields.

A KG is a form of data representation that consists of collections of descriptions of entities: events, concepts, or objects in the physical world where the entities are interconnected with each other via relations. It is a directed graph, with the entities as nodes and their relations as edges. Each directed edge in this graph, along with its head and tail entities, constitutes a triple, i.e., (head entity, predicate, tail entity). The description of entities has formal semantics, as provided by ontologies. As a result, machines can process information in KGs in an unambiguous manner. Major existing KGs include the Wikidata KG,^5^ the DBpedia KG,^6^ and the Google KG.^7^

KGQA systems have emerged as a prominent research topic in the KG community and have attracted massive attention. In particular, KGQA in the chemistry domain is a promising area of research, owing to the rapid growth of chemistry-related KGs and the potential advantages of a deep search of the chemical space. It is also critical to note that KGQA systems are fundamentally distinct from contemporary and widely adopted large language models (LLMs). LLMs are trained on extensive collections of textual data, primarily focusing on language modeling. In contrast, KGQA systems are designed to retrieve specific user-requested information from KGs, which are purposefully constructed to model factual aspects of the real world. Consequently, KGQA system outputs can be traced and lend themselves to easier verification. Thus, their divergent focus makes KGQA systems and LLMs distinct entities with unique objectives. In the context of chemistry-related research and industrial applications, which are the primary targeted application scenarios for KGQA systems, the feature of fact-oriented information retrieval (IR) plays a critical role. These domains require precise and accurate access to factual information regarding chemical compounds, reactions, properties, and other related data.

There are various implementation methods for KGQA systems. One such rule-based method is semantic parsing (SP) which transforms natural language queries into machine-readable logical forms that can be processed by KGs. In the past, chemistry KGQA systems such as Marie^8^ utilized a template-based SP method to interpret questions and generate SPARQL queries by filling query templates with internationalized resource identifiers (IRIs) for question-answering. The two key advantages of such template-based SP methods lie in their lower reliance on training data, allowing the system to be implemented quickly with limited training material and a higher accuracy of answers.

However, SP-based KGQA systems are faced with several limitations due to the increasing complexity of constructing precise queries, making them prone to errors. The chemistry domain consists of nonshallow ontologies, such as PubChem and OntoSpecies, necessitating multiple steps in a SPARQL query to retrieve the correct answer, contributing to the increasing difficulty of the QA system to construct an appropriate query. The nature of formal representations, such as SPARQL queries, makes them vulnerable to semantic or syntactic errors, which can lead to inaccurate results or query failure. Furthermore, the chemistry domain has multiple ontologies with different schemas, which creates scalability and robustness issues for SP-based KGQA systems, which are incapable of handling the heterogeneity of such KGs. Hence, there is a need for a KGQA system that can overcome the aforementioned challenges.

In order to bridge this gap, this paper explores IR-based KGQA systems,^9–11^ which have become popular due to their ability to generate limited answer candidates and rank them in accordance with the question, thereby overcoming the constraints of template-based SP approaches.

The purpose of this article is to present Marie and BERT, an IR-based KGQA system designed to facilitate extensive exploration of the chemical space. The proposed design of Marie and BERT aims to handle heterogeneous multiontology KGs and alleviate the costs of creating SPARQL templates by using hybrid KG embeddings. It aims to recognize entity references within natural language questions through a BERT-based entity linking (EL) model. The system also proposes the application of a predicate prediction model to ascertain the relationship within the question. Furthermore, the system is designed to include a score alignment model to handle multiple answer candidates from various Chemistry KGs. In order to address the issues posed by complex nonshallow ontologies, this paper proposes an implicit multihop relation derivation mechanism to create triples for training KG embedding models. Further, Marie and BERT aim to learn and infer information from the existing knowledge in the KG. It aims to invoke semantic agents that operate over TWA KG to perform dynamic calculations. Finally, the system intends to handle a vast number of chemical reaction mechanisms utilizing a combination of SP with the linked data fragment (LDF) when addressing chemical reaction-related questions. An online demonstration is available at this link https://como.ceb.cam.ac.uk/people/mab999/.

## 2 Related Work

### 2.1 World Avatar KG

The modern world is composed of intricate and complex systems, such as industrial symbioses, chemical plants, and cities. These systems are made up of diverse components, such as power generators, storage tanks, and abstract industrial operations. However, integrating the relevant data, knowledge, and models from these components to achieve complex tasks such as running simulations and optimizations and coordinating multiple components poses a challenge due to communication friction resulting from the use of heterogeneous conventions across domains.

TWA project is aimed at creating a comprehensive virtual representation of the physical world, with the goal of facilitating seamless integration and interoperability across diverse domains. The project builds on the concept of Digital Twins, which involves creating virtual representations of entities in industrial processes and takes it to the next level by extending it to cover all aspects of the physical world. The aim is to enable uniform integration not only between devices but also between devices and operations, thereby upgrading the internet of Things to the internet of Services.

Through the J-Park simulator (JPS),^12^ one specific implementation of the TWA project, a data management common ground for these components, has been provided to enable semantic interoperability. The JPS is now fully integrated into the TWA KG, which is a large-scale, dynamic KG (dKG) that integrates multiple ontologies from different domains. By using Semantic Web technology, information is represented in a machine-readable way, where concepts, entities, and the relations between them are formally defined and connected. This connection enables the retrieval and navigation of related data within a KG and interconnects previously isolated data sets by linking knowledge from different domains.

To update and maintain the large-scale KG over time, a number of agents are part of TWA KG, which perform functions such as data retrieval, simulation, and data update on top of the knowledge layer. In the TWA KG, agents are crucial for its dynamic functionality. These agents are web services deployed in a distributed manner and can be accessed via HTTP requests. Their semantic descriptions are stored in the KG, and they are semantically described by OntoAgent.^13^ This allows for the implementation of an agent composition framework, which enables the automated discovery, composition, and invocation of agents. The TWA KG hosts a wide range of agents, including the Thermodynamic Data agent (STDC agent) and the power conversion efficiency agent (PCE agent), which are specific to the chemistry domain.

### 2.2 Chemistry Ontologies

Marie and BERT is a KGQA system developed for chemistry that operates on top of the chemistry ontologies in TWA KG and the Wikidata KG.

#### 2.2.1 TWA Chemistry Ontologies

TWA KG is a large-scale, cross-domain, and dKG that follows linked data principles and integrates ontologies from various domains, including chemistry. TWA integrated and interconnected a number of ontologies specifically developed for representing chemical data such as OntoKin,^14^ OntoCompChem,^15^ OntoSpecies,^16^ and OntoMOPs^17^ ontologies.

OntoKin is an ontology that represents chemical kinetic reaction mechanisms. These mechanisms involve a set of stoichiometric reactions among different chemical species, described through various thermodynamic and transport model concepts and identified by OntoSpecies IRIs.^14^ It employs description logic (DL) to provide a semantic representation of chemical data within reaction mechanisms, which offers various benefits, such as interoperability between chemical kinetic systems, automated comprehension of chemical mechanisms by agents, and the capability to perform complex semantic queries on mechanisms in a web environment.^18^ OntoKin includes specific details about where the reaction takes place, such as in gas or on a surface, and offers a range of common reaction rate models for gas-phase and surface reactions. Additionally, OntoKin models the reversibility of a reaction using the reaction order and allows for easy detection of inconsistencies in thermodynamic, transport, and reaction data across mechanisms.

OntoCompChem is an ontology designed to enhance the semantics of chemical data in computational chemistry calculations, with a focus on density functional theory (DFT) for molecular systems.^19^ It extends the Gainesville Core and CompChem ontologies and employs DL-based semantics to enable interoperability between quantum chemistry software, reduce computational resource consumption via calculation reuse, and aid automated agents in understanding such calculations. OntoCompChem represents various aspects of a calculation, including its objective, software, theoretical level, charge, spin polarization, calculated Frontier orbitals, and self-consistent field energy. It also stores optimized geometries and computed vibrational frequencies for geometry optimizations and frequency calculations, linking them back to their corresponding calculations.

The OntoMOPs ontology reflects concepts and relationships relevant to the rational design of metal–organic polyhedra (MOPs).^17^ These concepts involve chemical and spatial factors that are used in chemical and spatial evidence-based reasoning, respectively. MOPs are assemblies made of organic and metal-based chemical building units (CBUs) that resemble regular polyhedra. To facilitate the design of new MOPs, OntoMOPs encode assembly models (AMs) and generic building units (GBUs) as mental blueprints that guide the selection of CBUs from available sets. Additionally, OntoMOPs employ the OntoSpecies ontology to instantiate the CBUs as species.

OntoSpecies^16^ is a fundamental chemistry ontology in TWA KG. This ontology contains about 36,000 IRIs and is constantly growing. The ontology also covers the basic chemical and physical properties of species. It is used as a foundation and has been expanded to encompass a diverse collection of identifiers, classifications, and uses of chemical species, as well as spectral data, in addition to information indicating its origins and attribution.

#### 2.2.2 Wikidata Chemistry

The Wikidata chemistry ontology is a subset of the Wikidata KG,^5,20^ focused exclusively on chemical species. This ontology was created by generating a customized dump that includes only instances classified under the subclasses of the class “group or class of chemical substances”. The Wikidata chemistry ontology currently comprises 33,061 distinct chemical species and approximately 50,000 triples.

The Wikidata chemistry ontology primarily captures chemical and physical properties of the included species, such as “mass”, “chemical structure”, “chemical formula”, “refractive index”, “ionization energy”, and “autoignition temperature”. In addition, it also includes the identifiers of these species in other databases such as PubChem compound identifier (CID), ChemSpider ID, and CAS Registry Number. This feature allows for easy linking and integration of Wikidata chemical data with external databases, facilitating cross-database searches and interoperability.

### 2.3 KGQA System

KGQA^2^ is a major focus in both the KG community and the natural language processing (NLP) community, as KGs are rich sources of semantic and structured data, and question answering represents one of the biggest challenges for NLP. In general, KGQA systems answer questions using two methods:^21^ the SP-based method^1,4^ and the IR-based method.^9,11^

#### 2.3.1 Semantic Parsing

In the SP-based method, questions are parsed into formal representations such as SPARQL queries,^22^ λ-DCS,^23^ or FunQL.^24^ These formal representations are then executed against the KG to find answers.^25–27^ There are three different approaches for SP-based methods: ranking methods, coarse-to-fine methods, and generation methods.

##### 2.3.1.1 Ranking Method

In the ranking method, the QA system first generates a list of formal candidate representations through a process known as candidate enumeration. For example, Bast and Haussmann,^25^ Berant and Liang,^26^ Abujabal et al.^27^ create candidate queries by filling prespecified query or machine-generated query templates with IRIs and other specific arguments. Another approach for candidate enumeration is to traverse the relation paths and neighbors linked to the topic entity in the question and add query operations including constraints or aggregation functions to form candidate queries.

Once the candidate queries are formed, the ranking method employs semantic matching techniques to select the top-ranked candidates. Neural models such as CNN,^28^ LSTM,^29^ and pretrained language models (PLM)^30,31^ are commonly used to score question–query pairs.

##### 2.3.1.2 Coarse-to-Fine Method

The coarse-to-fine method comprises two steps.^32–36^ First, the QA system predicts a rough skeleton that focuses on only the high-level structure of the query. Recent works often predict rough queries using encoder-decoder models. For example, Ravishankar et al.^32^ utilized a transformer-based SEQ2SEQ model initialized with BERT to generate the SPARQL skeleton corresponding to the question text. Sun et al.^4^ used a pipeline of subtasks, including question split and span prediction, for skeleton parsing. Das et al.^33^ used the pretrained T5 encoder-decoder model to directly produce a coarse skeleton.

With the rough skeleton in place, the QA system then populates it with details and creates the final queries. For example, Ding et al.^34^ used an attention-based BiLSTM network to link possible entities and relations in the questions and came up with all combinations of entities and relations for each skeleton. Hu et al.^35^ used BERT as a binary classifier to map attributes in the question to semantic relations.

##### 2.3.1.3 Generation Method

The generation method features two paradigms: graph search and encoder-decoder. In the works that leverage a graph search, formal representations are created by traversing the graph from the topic entity in the question. For example, Lan et al.^36^ iteratively searched through reachable relation paths starting from the topic entity and ranked the relation paths in the context of the question. The encoder-decoder paradigm aims to directly translate the questions to formal representations. Numerous works^37–40^ experiment with various encoder-decoder models to improve their correctness and well-formedness.

#### 2.3.2 Information Retrieval

The IR-based method, given a question, extracts a question-specific subgraph, which ideally includes all entities and relations related to the question, and applies a ranking algorithm to find the answer within the subgraph.^41–43^

One of the most prevalent IR-based KGQA methods is the KG embedding-based method. First, all entities and relations are represented within a vector space where their semantic relations are preserved. With the KG embedding, the likelihood or relative likelihood of a triple (*h*, *r*, *t*) can be measured by a scoring function . In a KG embedding-based KGQA system, the system translates the question into an embedding vector which serves as the relation embedding . Meanwhile, the system typically extracts an entity as the head entity and looks up its embedding . In addition, all neighbors within an n-hop distance from the head entity are extracted as candidate answers *A* = {*a*_1_, ..., *a*_*n*_}. To rank all candidates, for each *a*′ ∈ *A*, the likelihood of the hypothetical triple, ϕ(*h*, *r*, *a*′) is calculated.

Huang et al.^41^ first proposed the KG embedding-based method, which leverages KG embedding to answer simple questions. BiLSTM is used to convert the question into a vector representing the predicate, while TransE and TransR are applied to embed the KG. Saxena et al.^3^ then improved the KG embedding-based method by using the RoBERTa model for question embedding and applying the Complex embedding method for KG embedding. Shang et al.^42^ further improved the method for answering time-sensitive questions with temporal KGs.

The KG embedding-based method has been applied in several domain-specific KGQA systems. For instance, in the biomedical field, Rao et al.^43^ proposed a KGQA system over the Hetionet data set, which leverages RoBERTa and BioBERT for question embedding and applies Complex embedding for KG embedding.

### 2.4 Entity Linking

EL is a crucial component of KGQA systems, as it bridges the gap between human language and KG representation. EL involves identifying entity references, called “mentions”, in unstructured text and mapping them to corresponding entities in the KG. Traditional EL approaches use a pipeline structure consisting of three subtasks:^44^ named entity recognition (NER) for identifying the mention boundaries in text, Candidate Generation for generating prefiltered candidate lists for mentions, and Entity Ranking for ranking the candidates based on certain criteria.

Recent studies have demonstrated that PLMs like BERT^45^ significantly outperform traditional rule-based systems in EL.^46^ Wu et al.^47^ used BERT-based encoders in a two-stage fashion for EL, achieving state-of-the-art (SOTA) performance in local settings, while Yamada et al.^48^ considered global contextual information and achieved the highest SOTA performance in global settings. These studies highlight the advantages of using pretrained BERT for EL. However, most of these studies evaluate their approaches exclusively on general-topic KGs such as DBpedia^6^ or Freebase,^49^ limiting their application to laboratory setups instead of actual KGs in operation. Further research is needed to transfer these methodologies to specific scientific disciplines, such as chemistry, or specific downstream applications, such as QA.

EL for QA requires a specific approach since it involves short questions that have less context information compared to general EL, which focuses on lengthy and properly formed documents such as Wikipedia pages or news articles. Li et al.^50^ leveraged BERT-embedding for EL and designed it specifically for QA scenarios. However, their evaluation is based on DBpedia entities, limiting their study’s applicability to scientific domains. The vocabulary sets and domain-related characteristics of scientific domains present additional challenges that must be addressed.

The biomedical domain has presented some experience in EL, with the community developing BERT models based on domain-specific corpus, such as BioBERT^51^ and ClinicalBERT^52^ in addition to the original BERT models trained on Wikipedia and BookCorpus.^53^ Ji et al.^54^ fine-tuned pretrained BERT models for the sentence pair classification task and achieved a SOTA performance, demonstrating the advantages of using domain-adapted BERT models over the original BERT model. Chen et al.^55^ proposed a simpler model with a specific feature suited for biomedical terms. However, these approaches primarily focus on disease terms and clinical records, which are not directly applicable to the chemical QA scenario.

### 2.5 KG Embeddings for KGQA

KGs are widely used to represent structured information in various domains. One of the key challenges in working with KGs is to effectively manipulate and reason with the large amount of data they contain. KG embedding is a popular technique that can address this challenge by representing entities and relations in a continuous, low-dimensional vector space. This technique is widely used in tasks such as KG inference, relation prediction, KG completion, and in supporting KGQA.

A KG is represented as a set of triples , where each triple contains a head entity *h*, a tail entity *t*, and a relation *r* between them. The sets of entities and relations are denoted as *N* and *M*, respectively. To learn the embeddings, the loss function *L*(*h*, *r*, *t*) is defined, and the embeddings are iteratively updated to minimize the loss.

Over the years, a wide variety of KG embedding algorithms with unique loss functions have been developed, each capable of capturing different features of the KG. Choosing the appropriate embedding algorithm is crucial for achieving optimal efficiency and accuracy in embedding the KG, as the scale and structure of the KG can vary widely across domains.

#### 2.5.1 TransE

TransE^56^ is one of the simplest and most effective embedding methods for KGs that have only 1-to-1 relations. For a triple , it models the process of predicting the tail entity *t*, given the head entity *h* and the relation *r*, by translating *h* to *t* via *r*.

Given a triple , the TransE embedding in  results in three *d*-dimensional vectors denoted as . The TransE embedding is constructed such that  if (*h*, *r*, *t*) holds. To achieve this, the TransE score function  is introduced, which measures the distance between the sum of the vectors of the embedded head and the embedded relation and the embedded tail entity, defined as
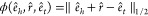
1

The training of TransE embedding is done by a pairwise method, which creates a fake triple  for each  by replacing the head or tail entity with a random head or tail. The loss function *L* is a margin ranking function, which takes the embeddings of both the true and fake triples,  and , as inputs and calculates the difference between the scores of the triple pair with a margin γ, defined as
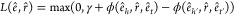
2

The training algorithm updates the embedding of all entities *ê* ∈ *E*_e_ and *r̂* ∈ *E*_r_ with respect to the gradient of *L*(*ê*,*r̂*) iteratively to minimize *L*(*ê*,*r̂*).

#### 2.5.2 TransEA

TransEA^57^ is an extension of the TransE embedding method that can handle numerical literals in addition to entity triples, . Some KGs, such as Wikidata, contain both entity triples and attribute triples, , where *l* is the attribute and *v* is a literal value associated with the entity. In TransE, attribute triplets are ignored.

In the TransEA method, a loss function for the embedding of numerical literals, *L*_l_(*ê*, *l̂*, *b̂*) is added to the TransE loss function for entity triples, *L*_r_(*ê*, *r̂*). For the embedding of a triple with a numerical literal , the loss function is defined as
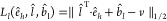
3where  is the embedding of the attribute *l*,  is the bias for the attribute , and  is the numerical value of the entity attribute.

The TransEA loss function *L*_r_(*ê*, *r̂*) for an entity triple (*h*, *r*, *t*) is identical to that of TransE. To model both entity triples and attribute triples, TransEA sums the two loss functions with an adjustable factor α. The combined loss function *L*(*ê*, *r̂*, *l̂*, *b̂*_*l*_) is defined as
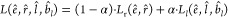
4

#### 2.5.3 ComplEx

ComplEx^58^ is a tensor factorization approach that represents the entities and relations in the KG in the complex space as complex-valued vectors, which enables the use of complex algebra to model semantic relationships among them.

For each triple , ComplEx generates  and defines a scoring function
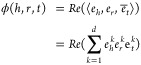
5such that ϕ(*h*, *r*, *t*) > 0 for all true triples, and ϕ(*h*, *r*, *t*) < 0 for false triples. The real and imaginary parts of the embeddings capture different aspects of the semantics of the entities and relations, respectively, and their combination in the scoring function allows for the modeling of more complex and nuanced relationships among them.

#### 2.5.4 TransR

TransR^59^ is an extension of TransH,^60^ which encodes entities and relations into distinct spaces. For each triple (*h*, *r*, *t*), entity embeddings are  and relation embeddings are . A projection matrix  is learned for each relation *r* that can project an entity to different relationship semantic spaces.^59^ Each of these spaces captures a different aspect of an entity that is related to a distinct relationship. The projected vectors of entities are denoted as
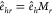
6
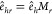
7

The loss function is defined as
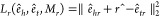
8

## 3 Data Sets

### 3.1 EL Data Set

To train an effective EL model, a high-quality EL data set is required, which consists of text labeled with mentions and their corresponding IRIs. The EL data set is designed to support the training of EL models that can recognize mentions of these entities in question and link them to their corresponding IRIs in the KG. The EL data set focuses on several domains, including chemical formulae, names, SMILES, and InChI, as well as various classes such as MOPs and CBUs.

The data in the EL data set is collected from Wikipedia pages related to chemistry and chemical species. These pages contain not only textual information about the chemical species but also their Wiki IDs. By utilizing the information provided by Wikidata, we map these Wiki IDs to their corresponding PubChem CIDs.

For data not available on Wikipedia, such as classes, e.g. MOPs and CBUs, from a specific domain ontology, we employ scripts to automatically generate training data from the ontology. These scripts traverse the domain ontology, gathering IRIs and labels. Subsequently, the scripts generate text with labeled mentions by applying predefined templates and rules.

The input to the data set is text labeled with mentions, and the output is the IRI of the mention.

### 3.2 Relation Prediction Data Set

The relation prediction data set is designed to train a model for predicting relation embeddings given a question. The purpose of this data set is to enable the development of models that can accurately predict relation embeddings between entities in a KG based on the input question.

The input to the data set is a question, and the output is the relation embedding. The data set covers both explicit and implicit relations within all chemistry ontologies in this project’s scope, providing a comprehensive range of relation types to support the development of relation prediction models.

The data for this data set are also generated using scripts. These scripts traverse the KG and gather both explicit and implicit relations along with their labels. Predefined question templates are then populated with the relation labels to generate questions. For instance, a question template might be “how much does it weigh?” where the entity is omitted. Implicit relations are prelabeled manually before the data generation process.

### 3.3 Score Alignment Data Set

The score alignment data set is designed to train a model for reranking the answers returned by different domain question answer (QA) engines based on the input question and the ontologies with which it is affiliated. The purpose of this data set is to enable the development of models that can accurately align scores across different QA engines and improve the accuracy of question answering.

The input to the data set is a question and a list of ontologies with which the question is affiliated. The output is a reranked list of answers returned by different domain QA engines based on the alignment of their scores. The data set covers possible questions from all chemistry ontologies within this project’s scope, providing a comprehensive range of question types to support the development of the score alignment model.

The training data set is created using a method similar to that employed for the relation prediction data set.

## 4 Design

### 4.1 Overview

**Figure 1:**
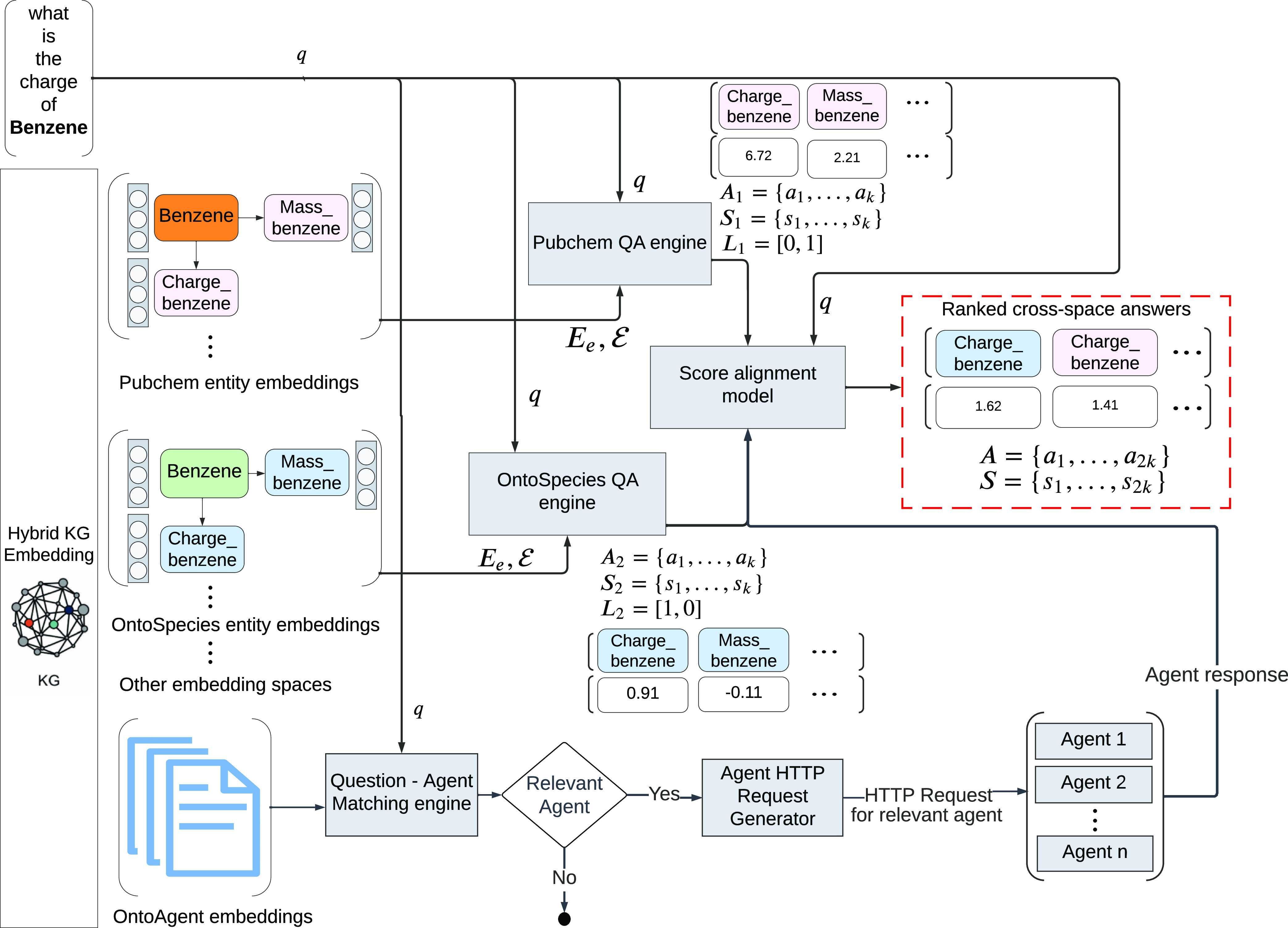
Overall design of the Marie QA system. For each ontology, an independent set of entity embeddings is trained, where *E*_e_ is an indexed set of entity embeddings and  is the indexed set of entity IRIs, and an independent QA engine is implemented. Given a question *q*, each QA engine will return a set of answers together with their scores *S* and the encoded label *L* of the corresponding embedding space *E*_e_. Given the scores *S* and the embedding space labels and the question *q*, the score alignment model will adjust the scores with respect to the inputs and rerank the answers returned from different embedding spaces and QA engines.

The cross graph question answering (CGQA) engine, as depicted in Figure 1, serves as the primary interface for the QA engines developed for each ontology within the Marie and BERT system. The CGQA engine incorporates seven distinct QA engines, with each one designed for a specific chemistry ontology in the TWA KG. Each QA engine operates on the KG embeddings associated with its particular ontology.

When the CGQA engine receives a natural language question, it distributes the query to all QA engines that operate in parallel. In response, every QA engine generates a collection of answer candidates and ranks them by using a score function. This function assesses the likelihood of each candidate answer’s existence.

Subsequently, the CGQA reranks the answers provided by the individual QA engines and presents a final set of the most suitable answers within its context.

### 4.2 Entity Extraction

**Figure 2:**
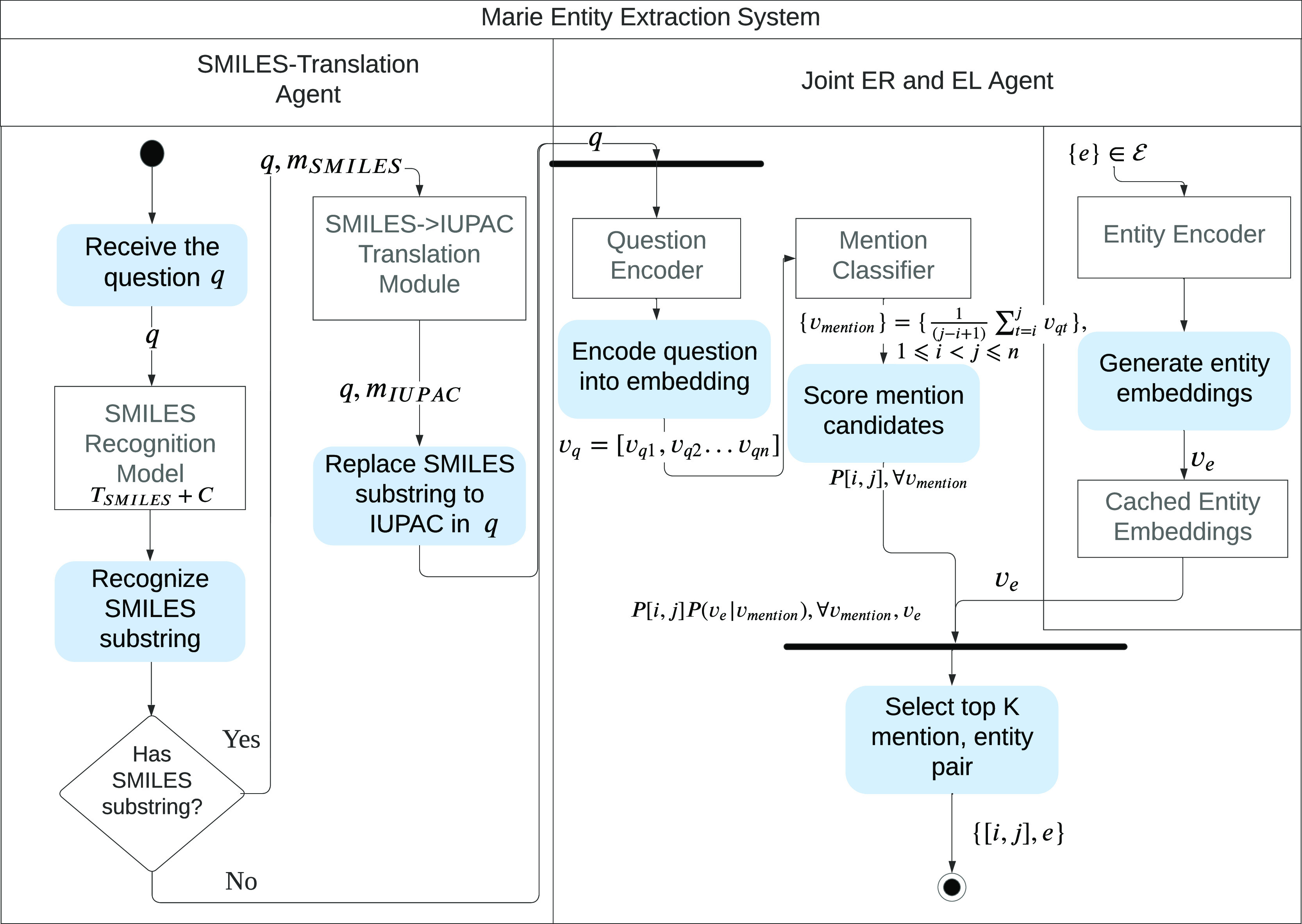
Workflow of the Entity Extraction module is completed in two stages. Left: SMILES-translation stage. If a SMILES substring exists in the question *q*, it is identified and translated through an off-the-shelf SMILES to an IUPAC translation module. The question is then preprocessed by replacing the SMILES string with its IUPAC translation. Right: joint ER and EL stage. Token-level embedding of the question text; *v*_q_ is obtained from the question encoder. The mention classifier then outputs the possibility of a mention span [*i*, *j*] in the question being the true mention span, *P*([*i*, *j*]). Its embedding is the average embedding of the tokens in the span, *v*_mention_. Entity embeddings *v*_*e*_ are generated by the Entity Encoder and cached before runtime. *P*(*v*_e_|*v*_mention_) is the probability of an entity being the gold entity given a mention. The mentioned entity pairs are eventually scored based on the joint probability *P*[*i*, *j*]*P*(*v*_e_|*v*_mention_).

It is crucial to first obtain the IRI of the head entity referred to in the input question before a QA engine can predict the answers. For a KG , a set of entities  and question text *q*, the Entity Extraction module aims to find the corresponding IRI *h* of the head entity  referred to in *q*. The occurrence of an entity in a natural language sentence is referred to as a “mention”. The conventional workflow involves first identifying a mention span in the question and then scoring entities based on their similarity to the mention span text. This process is divided into two steps: ER, followed by EL (Figures 1 and 2). However, we propose a different workflow for three main reasons:

1. Mention boundaries of entities are irrelevant in question answering.
2. Scientific notations like SMILES differ significantly from natural language, making them difficult to process with a single unified language model.
3. A stand-alone ER system infers solely from the question text and does not utilize the information in the KG. Once the span is marked, additional information in the question text is discarded and not utilized in the follow-up EL. Moreover, with the question text being short and lacking context, ER as a standalone step might be error-prone and become the bottleneck.

Instead, we implement a joint ER and EL approach similar to ref (50). Additionally, we include a SMILES translation module to extract any SMILES names and convert them to IUPAC names, which are more accessible for a language model to make predictions. We propose the following two-stage workflow, as shown in Section 2.

#### 4.2.1 SMILES Translation

We recognize SMILES names as unique surface forms in the chemical domain that are difficult to process with a unified language model. Consequently, we designed a SMILES to IUPAC name translation module as a preprocessing step. For this purpose, we fine-tuned a *BERT*_BASE_ model *T*_SMILES_ along with an additional linear classifier *C* on the token classification task, following standard practices. Each token in the question text is classified as belonging to a SMILES string or not. The training procedure is detailed in Section 9.

Given the question text *q*, the trained model can now identify SMILES substrings. Once identified, we translate the SMILES string to its common IUPAC name and replace it in the question text before feeding it into the Joint EL and ER model for entity extraction. For translation, we employ the off-the-shelf library STOUT.^61^

#### 4.2.2 Joint ER and EL

A joint ER and EL system considers all possible spans of the question text as mention span candidates and measures the joint probability of a span being the mention span and the mention matching a candidate entity in the KG. The design for the model follows the method of ref (50). The model consists of three trainable components.

1. The question encoder, which encodes the question text.
2. The entity encoder, which generates entity embeddings from the KG.
3. The mention classifier, which outputs the probability of all mention spans being the true span.

The model is trained using the data set described in Section 3.1. We follow the methodology in ref (50) and train the model in a two-step fashion. First, we use the EL data set with the given mention spans to train a plain biencoder. Then, we freeze the entity encoder to train the new question encoder and the mention classifier for the joint ER and EL task. Details of the training procedure can be found in Sections 7 and 8. After the training is completed, the models are frozen, and the entity embeddings for all KG entities are cached for faster computation.

During runtime, a question is first fed into the question encoder to obtain question embedding. Using question embedding, the mention classifier outputs the probability of each mention span being the true span. Given a candidate mention span, the mention embedding is calculated as the average of the textual embeddings of all tokens in the mention span. The probability of each entity being the true entity given this mention span is then calculated by comparing the mention embedding and the question embedding.

The final joint probability of an entity-mention span pair is computed as the product of two probabilities: the probability of the mention span being the true span and the probability of the entity being the true entity given this mention span. For all entity-mention span pairs, we select the top *K* pairs with the highest joint probability.

### 4.3 Hybrid KG Embeddings

The KG embedding module aims to represent entities and relations within a KG in vector space. Several KG embedding methods are available for KGs of varying complexity and structures. Due to the heterogeneous and cross-domain nature of the TWA KG, we implemented a novel system wherein different ontologies are embedded using distinct embedding methods.

In addition, to handle both 1-to-*N* relations and numerical attributes, we implemented a new embedding algorithm named TransRA, which jointly embeds numerical attributes based on the TransR algorithm. The projection function is identical to TransR. The loss function of this embedding method is defined as
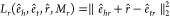
9
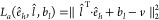
10where  is the embedding of the attribute,  is the bias for the attribute *l*, and  is the numerical value of the entity attribute.
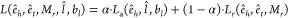
11

The training pseudocode of TransRA is defined in Algorithm.

Including TransRA, the candidate embedding methods for embedding the KG include TransE, TransEA, TransR, TransRA, and Complex embeddings.

OntoSpecies, Wikidata chemistry, and OntoMOPs contain 1 to *N* relations, in which one head node can connect to multiple tail nodes via the same relation. The majority of the information contained in these ontologies is numerical rather than semantic.

Consequently, the TransRA embedding method is chosen for its ability to model 1 to *N* relations, compared to the TransE embedding method.^59^ Additionally, experiments conducted to analyze the performance of different embedding methods on the task of inferring knowledge from OntoSpecies revealed two main findings. First, the addition of joint numerical embedding significantly improves the inference performance on OntoSpecies, which is primarily numerical. Second, the experimental results also demonstrate that joint numerical embedding is significantly less effective when applied to Complex embedding.

As a result, the TransRA embedding method is selected as the embedding method for OntoSpecies, Wikidata chemistry, and OntoMOPs. OntoCompChem and OntoKin contain less numerical data. However, these two ontologies have nonshallow structures, where the answer nodes can be multiple relations away from the head node. Therefore, Complex embedding is chosen for its capability to capture implicit relations.^58^

#### 4.3.1 Implicit Multihop Relation Derivation

Ontologies like OntoCompChem are nonshallow and have complicated structures, as illustrated in Section 3. In these ontologies, a property of a species can be three or more hops from the species itself. Consequently, the formal representation for querying such KGs can be complex and, therefore, prone to errors. This complexity also poses challenges for IR-based methods. Although Complex embedding can capture implicit relations, the complicated structure of OntoCompChem limits its capability and accuracy of relation prediction.

**Figure 3:**
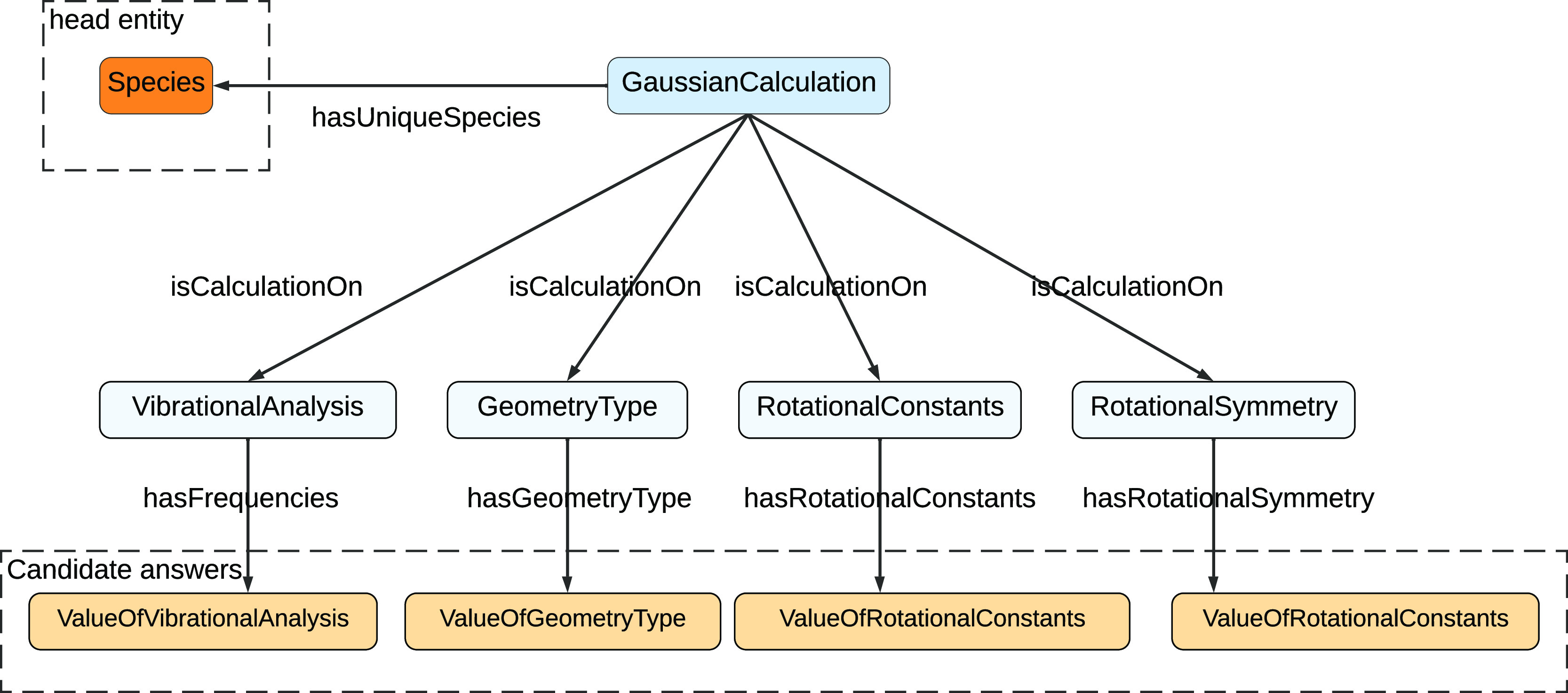
Illustration of the structure of the OntoCompChem ontology. As shown in this figure, the head entity, which is a species, is 3 hops away from the candidate answers. In addition, the first two hops from the head entity to the candidate answers are identical.

To address this issue, we adopt an implicit multihop relation derivation algorithm as described in the algorithm. This approach involves deriving possible implicit relations in advance and creating a limited set of triples with these implicit relations for training the KG embedding models. This method does not require significant effort but greatly enhances the accuracy of the relation prediction (Figure 3).

## 5 Implementation/Method

### 5.1 QA Engine Workflow

**Figure 4:**
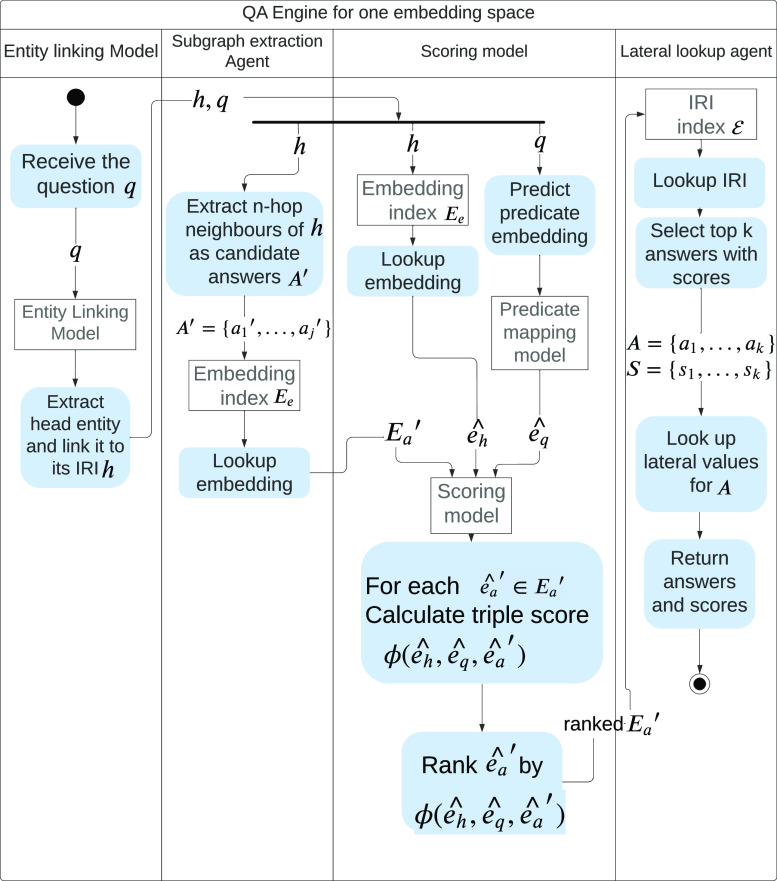
Working flow of the QA engine for one embedding space, where the indexed set of entity embedding is denoted as *E*_e_ and the indexed set of entity IRIs as . *q* represents the question string and  represents the predicted embedding vector of *q*, *h* denotes IRI of the head entity within the question while  denotes the embedding of *h* in the vector space, *A*′ denotes the IRIs of a set of candidate answers while  denotes the embeddings of *a*′ in the vector space. For each ,  is calculated to score the hypothetical triple .

Figure 4 illustrates the workflow of a QA engine operating on the embedding space for one KG, , where the set of all entity IRIs is denoted as , the set of all relation IRIs as , the embeddings of entities as *E*_e_, and the embeddings of relations as *E*_r_.

Each QA Engine receives a question string, *q*, as input. The question is passed to the EL module, which extracts the head entity in the question and links it to its IRI, *h*. The subgraph agent then extracts the candidate answers, , that are within *n*-hops from *h*. For each *a*′ ∈ *A*′, we look up its embedding to create , and similarly, we look up the embedding  of *h*.

In the meantime, given the question *q*, the relation prediction model maps the relation in *q* to a relation embedding, . For each candidate answer , the embeddings of a hypothetical triple  are created. All hypothetical triples are then scored using the scoring function  of the embedding method being used for . A set of the candidate answers embeddings  with the highest scores are formed and converted to their IRI forms *A* = {*a*_1_, ..., *a*_*k*_}, where *k* ≤ *j*. If *a* ∈ *A* is linked to numerical values, then the numerical values are retrieved by the lateral lookup agent.

### 5.2 Information Derivation

Inference in KGs refers to the process of deducing new information from existing knowledge represented in the graph. The use of inference can enhance the accuracy and completeness of the information represented in the graph and can be used for a variety of tasks such as link prediction, question answering, and recommendation systems.

The information derivation task can take several forms, such as tail, relation, or head inference. Marie and BERT employ the TransRA embedding methodology to achieve the inference task on OntoSpecies. The performance of TransRA compared to the other embedding methods is shown in Table 1.

**Table 1:** Performance of Different Embodiments on the OntoSpecies Tail Inference Task

	f_mrr	f_hit_1	f_hit_5	f_hit_10
TransE	0.4055	0.3396	0.4528	0.6038
TransEA	0.4821	0.3396	**0.6603**	0.7169
TransR	0.3911	0.2452	0.6038	**0.7547**
TransRA	**0.5134**	**0.3962**	0.6226	0.6981
complex	0.3736	0.1698	0.6603	0.6981
complex_numerical	0.233	0.1509	0.3396	0.415

Information derivation offers several applications. One potential application is to infer the use of a particular species represented in OntoSpecies.

### 5.3 Numerical Questions

Marie and BERT’s CGQA is capable of handling numerical questions in the chemistry domain. For example, they can answer questions like, “which species have a molecular weight less than 50 g/mol?” Based on the filtering criteria specified in such questions, such as “larger”, “smaller”, or “close to”, the set of candidate species is narrowed down by predicting their numerical attributes.

To handle numerical questions, the QA engine employs two rounds of filtering operations. In the first round, a set of candidate species is obtained based on their predicted numerical values, which must align with the numerical filter specified in the question. Next, the actual numerical attribute values for this subset of species are determined by using their embeddings. Finally, the second round of filtering produces a final list of answer candidates whose actual numerical attribute values adhere to the numerical filter in the question.

This methodology is time-efficient, which is crucial for effectively answering numerical questions. We evaluated the accuracy of the numerical filtering mechanism by evaluating its recall, precision, and F1 score. Two separate tests are conducted; the filtered test is conducted given the true relation and true numerical operators in the question, while the unfiltered test is conducted without the ground truth information. Table 3 shows the results of the two tests.

**Table 2:** Evaluation of the EL

model/test data set	template questions	SMILES questions
ChemData + FuzzySearch	0.21	
ours	0.72	0.37
ours w/o translation	0.72	0.055

**Table 3:** Accuracy of Numerical Filtering Questions

test	recall	precision	F1 score	operator accuracy	mean value error
unfiltered	0.8367	0.7177	0.7726	1.0	0.4583
filtered	0.8367	0.9684	0.8978	1.0	0.5519

### 5.4 OntoKin Chemical Reactions

The OntoKin chemical reactions ontology comprises 89,780 unique reactions, 9964 unique species, and 679,737 triples. Several experiments were conducted to embed this ontology; however, the embedding faces two major challenges. First, the large size of the ontology results in a high embedding cost. Moreover, upon analyzing the structure of the ontology, it becomes evident that all nodes within the ontology are connected, and no subgraph can be separated without breaking connections. Second, the ontology structure is highly imbalanced. Some very common species are connected to a significantly larger number of reactions compared with some rare species. For example, the species hydrogen is connected to 7316 reactions, whereas *C*_9_*H*_7_ is only connected to 76 reactions. Due to this imbalance, the embedding becomes less effective,^62^ which further complicates the embedding process for the OntoKin chemical reaction ontology.

**Figure 5:**
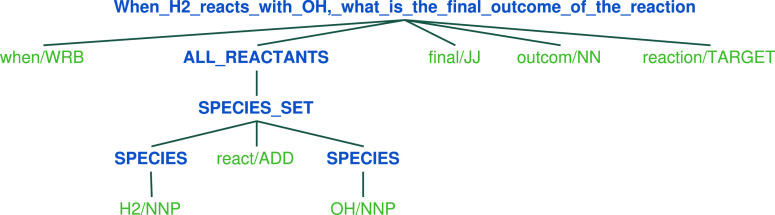
Grammatical dependency structure of the question “when H2 reacts with OH, what is the final outcome of the reaction”.

To address the aforementioned challenges, we adopted a more cost-effective SP-based approach for querying OntoKin chemical reactions. This approach involves the use of the NLTK SP module to analyze the grammatical dependencies in a question and setting up an LDF server to support reaction querying. The SP module leverages probabilistic dependency grammar to parse the question into a tree structure, illustrating the grammar dependencies among the different components within the question. We then identify the reactants or products within the question. Figure 5 illustrates an example of the grammatical dependency in a reaction query question.

Nonetheless, due to its large scale, hosting the ontology using a traditional graph database, such as Blazegraph or RDF4J, can be computationally expensive. The LDF^63^ approach offers a solution for querying semantic data. This method enhances the scalability and availability of the query end point through partitioning strategies and caching mechanisms. Consequently, we implemented an LDF server to host the OntoKin chemical reactions ontology.

### 5.5 Semantic Agents for Dynamic Calculations

**Figure 6:**
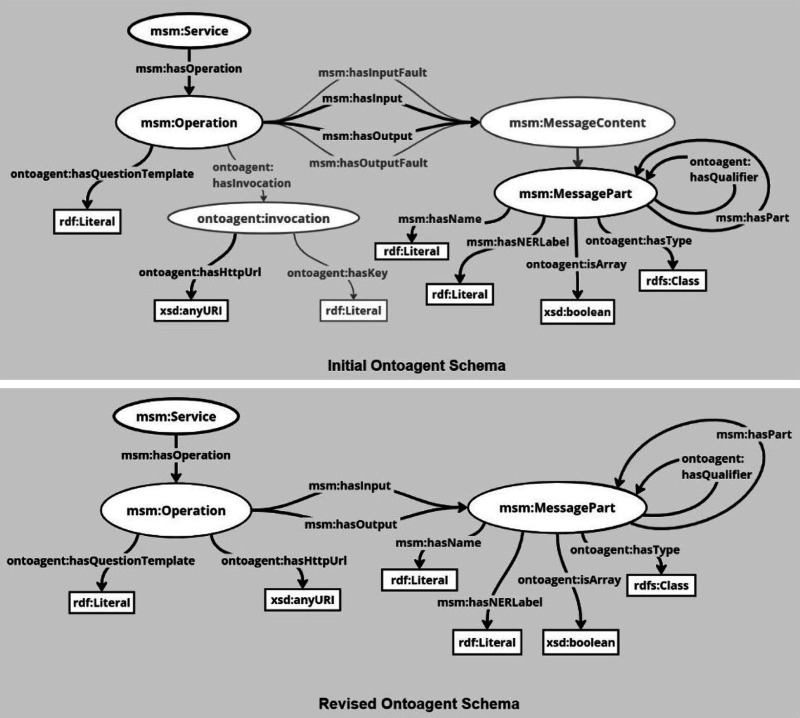
Revisions to the schema of the OntoAgent ontology used to instantiate the Agents and create their respective embeddings. The grayed-out sections in the Initial OntoAgent schema represent certain components in the schema that have been omitted to enhance clarity and eliminate redundancy.

In order to perform calculations on the fly, Marie and BERT access the dynamic components of the TWA KG: the agents. Agents are web services that are deployed in a distributed manner and accessible via HTTP requests, with their semantic descriptions stored in the KG. In TWA KG, an agent is semantically described using OntoAgent.^13^ The typical OntoAgent description of an agent has been revised and simplified to include a detailed description of its input/output (I/O) signatures, its URL, and associated question templates, as illustrated in Figure 6.

The TWA KG encompasses a wide range of agents. Within the chemistry domain, these agents consist of a thermodynamic data agent (STDC agent) and a PCE agent. The STDC agent calculates the gas-phase thermodynamic properties of a chemical species as a function of temperature *T* and pressure *P*,^64^ while the PCE agent computes the power conversion efficiency of an organic solar cell, given the SMILES string of the donor molecule of the cell.^65^

In the previous Marie QA system, the process of agent discovery relied on SPARQL queries, which are prone to errors. Additionally, using a simple classifier for agent discovery proved to be inadequate for distinguishing between very similar agents. To address these challenges, we have implemented an agent interface in the Marie and BERT CGQA systems that is independent of all QA engines. This agent interface operates on the OntoAgent embeddings for chemistry-domain agents, with concepts in the OntoAgent description embedded using TransR.

The embeddings of the input/output configurations for each agent are utilized to create matrices representing each semantic agent. Given a question, we employ a BERT-based relation prediction model to predict the relation embedding present in the question. In this approach, a pretrained BERT model is connected to two fully connected layers, which transforms the question into a relation embedding. Subsequently, we conduct similarity matching between this relation embedding and all available semantic agents, using their respective matrices to determine the question-agent affiliation.

Furthermore, we employ a chemical EL model to identify the key components within the question. For instance, in the question “what is the heat capacity of benzene at 100 K?”, the term “benzene” is labeled as “species”. This labeling is achieved through a fuzzy set search within a predefined vocabulary list containing a large number of instance and class labels, including species. The label with the highest similarity to the mention is then looked up in a predefined vocabulary dictionary, where labels are mapped to their types. For example, “benzene” is mapped to the type “species”.

Qualifiers, including numerical values and their units, are identified using regular expressions. For example, in the aforementioned question, the qualifier is identified as “100 K” and labeled as “temperature”. However, in the proof-of-concept implementation, we did not handle the unit conversion and assumed that all temperature units are in Kelvin (K) and pressure units are in Pascal (Pa).

If a suitable agent is identified, then the key components are passed to an agent invocation interface, which generates the HTTP request, invokes the appropriate agent, and returns the answer to the score alignment model for further processing.

### 5.6 Score Alignment Model

By design, the QA engine for each ontology is implemented separately using independent embedding spaces. However, there is also overlap between domains. For instance, both Wikidata chemistry and OntoSpecies can answer questions about the basic physical and chemical properties of some species. Consequently, the correct answers might come from multiple QA engines. Moreover, since different embedding methods are applied to the ontologies, the scale of the answer scores varies, rendering it impossible to directly compare the scores of answers originating from different QA engines.

To address this issue, we implemented a score alignment model that adjusts the scores from multiple QA engines, using the question and domains of the scores as inputs. The intuition behind this model is to predict a bias for the score associated with each answer based on the question-ontology affiliation and the domain from which the score is derived. Algorithm outlines the training process for this model.

Table 5 presents the results of an ablation test for the score alignment model. In the absence of the score alignment model, the set of scores from each ontology is normalized by dividing each score by the highest score within the set. Subsequently, the scores from different domains are reranked together. The results indicate that the score alignment model effectively improves the accuracy of the final answers.

**Table 4:** Evaluations of Separate QA Engines

	hit 1 rate	hit 5 rate	hit 10 rate	mrr
Pubchem	0.8122	0.8155	0.8155	0.8132
Pubchem filtered	0.9449	0.9544	0.9544	0.9996
Ontocompchem	0.3366	0.39	0.39	0.7971
Ontocompchem filtered	0.8147	0.9621	0.9621	0.8542
Ontokin	0.88	0.885	0.885	0.6767
Ontokin filtered	0.9943	1.0	1.0	0.8755
OntoSpecies	0.7907	0.8210	0.8210	0.7784
OntoSpecies filtered	0.9168	0.9519	0.9519	0.8920
Wikidata	0.4910	0.5731	0.5731	0.5255
Wikidata filtered	0.8182	0.9364	0.9364	0.8673
OntoMOPs	0.7356	0.8451	0.9132	0.8736
OntoMOPs filtered	0.8612	0.8933	0.9245	0.8952

**Table 5:** Ablation Test for Score Alignment

	hit 1 rate	hit 5 rate	hit 10 rate	mrr
w/o score alignment	0.4762	0.9452	0.9469	0.5677
with score alignment	**0.9822**	**0.9879**	**0.9903**	**0.9784**

## 6 Evaluation

This research focuses on a novel data set developed specifically for chemistry-related KGQA. The data set is tailored for the TWA chemistry KG, which is presently the sole known KG dedicated to chemistry. While benchmarking the performance of other KGQA systems against the Marie system is impractical due to the significant retraining costs involved, we have taken the initiative to share the scripts for creating this new data set on GitHub (https://github.com/cambridge-cares/TheWorldAvatar/tree/main/MARIE_AND_BERT). This availability enables future implementations to perform benchmarking and comparisons with other KGQA systems, fostering advancements in chemistry-related KGQA research. This research includes an evaluation of the individual components within the KGQA system to assess their performance. Furthermore, the overall performance of the KGQA system as a whole is thoroughly evaluated to gauge its effectiveness in retrieving accurate and relevant information from the TWA chemistry KG. A series of ablation tests is also presented.

### 6.1 Entity Linking

Due to the lack of available chemical QA data sets, we collected chemistry-related natural language questions from https://socratic.org/chemistry. However, the original questions exhibited a significant imbalance in the distribution of entities. To address this, we manually selected 172 template questions and generated our training and test sets by replacing the original mentions in the question templates with entity mentions sampled from the KG. These entities were randomly sampled from a combination of the first 5000 PubChem compounds and our chemical ontologies. For PubChem entities, the mention could randomly be the IUPAC name, chemical formula, or any of its aliases.

To demonstrate our model’s ability to understand unseen question structures, we ensured that the training and testing templates were separate and had no overlap. We trained our model on 20,000 questions generated from 32 templates and tested it on 5000 questions generated from 140 templates. For SMILES questions, we randomly selected 500 SMILES expressions from the PubChem compound list and generated questions from the 140 test templates. We measured the *K* – 1 accuracy of our predictions.

We employed a traditional pipeline denoted by ChemData + fuzzysearch as a baseline for evaluation. This pipeline first recognizes possible mentions using ChemDataExtractor,^66^ a popular toolkit for chemical ER using random fields combining custom dictionaries and rule-based NLP techniques. The extracted mention is then linked by a fuzzy name search, including IUPAC names, chemical formulas, and all known aliases.

To compare the performance of our translation pipeline to training with SMILES expressions directly, we trained a second set of models following the same procedure, except with an additional 4000 training questions generated from SMILES expressions. In this case, the extra translation module was not employed, and the SMILE expression was passed through and compared by the biencoder directly. The *K* – 1 accuracy of all settings is shown in Table 2.

### 6.2 QA Engine

In order to assess the accuracy of question answering, we first evaluated the QA engine implemented for each ontology using an evaluation data set specifically created for the ontology. Table 4 presents the evaluation results for each ontology, which include four metrics: hit 1 rate, hit 5 rate, hit 10 rate, and mean reciprocal ranking (MRR). The hit *n* rate indicates the percentage of instances in which the true answer is found within the top-*n* answers returned by the QA engine. MRR is defined as follows
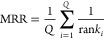
12where *rank*_*i*_ refers to the rank of the true answer and *Q* represents the total number of questions used for evaluation. The evaluation results presented in Table 4 reflect the specific data prepared for the evaluation, which have certain limitations. It is important to note that the data used for evaluation primarily consists of script-generated content, which inherently imposes constraints on its coverage and variety.

For each ontology, we calculated a filtered and unfiltered set of accuracy results. Filtered accuracy measurements were obtained by providing the QA engines with the true head entity, while unfiltered accuracy measurements were calculated without providing the true head entity. Consequently, due to errors in EL, the filtered accuracy results tend to be higher than the unfiltered accuracy results.

## 7 Results and Discussion

**Figure 7:**
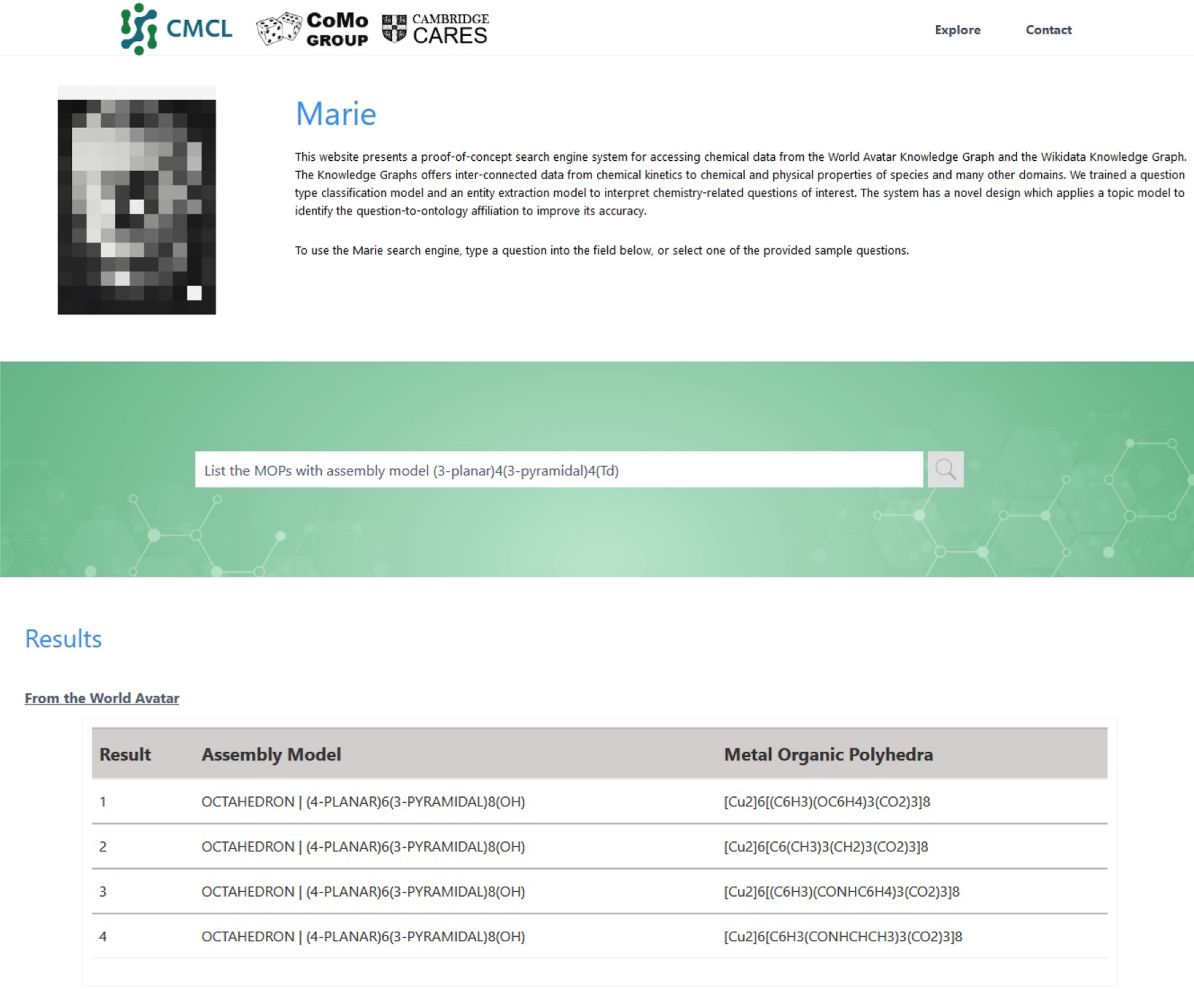
Response from Marie and BERT to the question “List the MOPs with AM (3-planar)4(3-pyramidal)4(Td).”.

**Figure 8:**
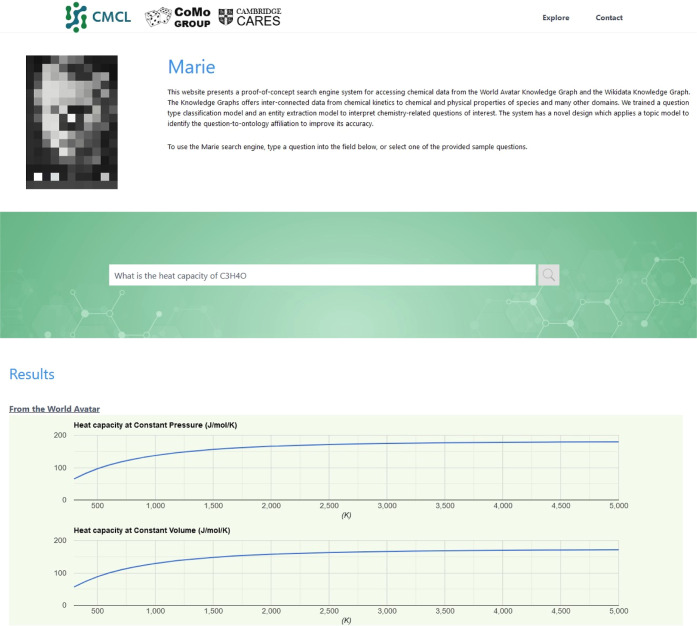
Response from Marie and BERT to question “what is the heat capacity of C3H4O”.

This section showcases the performance of Marie and BERT through a series of screenshots that provide a clear and insightful view of the KGQA system’s capabilities in producing accurate responses. For instance, Figure 7 demonstrates the ability of Marie and BERT to answer a question from the metal organic polyhedras (MOPs) domain. Figure 8 demonstrates the ability of the system to answer a question dynamically by invoking the thermodynamic data agent (STDC agent). The response includes a visual representation of the heat capacity of cyclopropanone at a constant pressure and constant volume.

Users can interact with Marie and BERT by following this link https://como.ceb.cam.ac.uk/people/mab999/. However, this system is still under development, and the accuracy of the results will increase with further refinement of the underlying ontologies.

## 8 Conclusions

In this paper, we introduced a novel IR-based KGQA system that employs hybrid KG embeddings to accurately answer natural language questions in the chemistry domain. Our system improves the robustness of chemistry KGQA by utilizing IR-based methods, which are less sensitive to errors than SP-based KGQA systems.

To address the heterogeneous structure of the TWA chemistry KG, we have applied different embedding strategies for various chemistry ontologies based on their complexity and structure. We have also proposed a score alignment model that reranks results from different embedding spaces by providing a scoring bias based on the question-ontology affiliation. This enables the production of a final list of interontology answers scored on a uniform scale.

In order to manage the large volume of numerical data in the TWA KG, we have combined the translation embedding method, TransR, with a joint numerical embedding model to form TransRA. This model enhances the quality of KG embedding and, consequently, improves the accuracy of the information derivation task. Additionally, we implemented a numerical filtering mechanism to reduce the time and space complexity of answer selection for numerical questions.

Furthermore, we have developed an implicit multihop relation derivation algorithm to handle nonshallow ontologies, such as OntoCompChem, which increases the QA accuracy. Marie and BERT are also capable of answering questions that require on-the-fly calculations by invoking semantic agents for dynamic calculations in a robust, scalable, and autonomous manner.

We have also implemented a cost-effective SP-based method to handle many chemical reactions, supported by an LDF service, and to answer questions related to chemical reactions.

Finally, our EL module is designed to identify SMILES strings and translate them into their respective IUPAC names. As language models such as BERT struggle with handling SMILES strings for similarity comparisons, this translation significantly improves the accuracy of identifying species represented by SMILES strings in a question.

However, the lack of a proper Chemistry QA data set that is diverse and varied presents a challenge. The task requires both chemistry domain knowledge and expertise and an understanding of the underlying KG structure, which poses difficulties.

Future work includes efforts to reduce the cost of training embedding models and to make the system more robust. Expanding the system to integrate the knowledge of other domains could also be a fruitful avenue for future research.

## Data Availability

The training data, evaluation data, and evaluation results are available in the GitHub repository https://github.com/cambridge-cares/TheWorldAvatar/tree/main/MARIE_AND_BERT. All of the third-party software used in this system is free and available. The Python environment suitable for operating the QA system is Python3.7, and all Python libraries required and their versions are listed in the file “requirements.txt”. All of the packages from NLTK 3.5 also need to be downloaded and installed. A docker solution is also available for quick deployment using “Dockerfile”. The latest version of Docker Desktop is required.

## Appendix: Notations and Algorithms

Table A1 explains the notations used herein.

**Table A1:** Explanation of Notations

notation	explanation
	indexed set of all entities in in IRI forms
	indexed set of all relations in in IRI forms
= {(*h*, *r*, *t*): *h*, *t* ∈ ,	a knowledge graph
(*h*, *r*, *t*)	a triple in the form of IRIs
(*ê*_*h*_, *r̂*, *ê*_*t*_)	a triple in the form of embedding representations
*n*	the total number of entities in
*m*	the total number of relations in
*d*	dimension of the embedding representations
*q*	the question in text
*E*_r_ ∈	indexed embedding representations of all predicates in
*E*_e_ ∈	indexed embedding representations of all entities in
*ê*_*q*_ ∈	predicted relation embedding
*ê*_*a*_^′^ ∈	embedding of a candidate answer for *q*
*j*	number of entities within three hops distance from *h*
*A*′ = {*a*_1_^′^, ..., *a*_*j*_^′^}	entities within three hops distance from *h*
*E*_*a*_ = {*ê*_*a*1_, ..., *ê*_*aj*_}	embeddings of entities within three hops distance from *h*
*k*	number of final answers
*A* = {*a*_1_, ..., *a*_*k*_}	top *k* answers in IRI forms ranked by their scores
ϕ(*ê*_*h*_, *ê*_*q*_, *ê*_*a*_^′^)	scoring function for a triple
η	learning rate for model training
*L*(·)	loss function
⊙	element-wise product, e.g., [*a*_1_ *a*_2_]⊙[*b*_1_ *b*_2_] = [*a*_1_*b*_1_ *a*_2_*b*_2_]
̑	concatenation operator, e.g., [*a*_1_ *a*_2_] ̑[*b*_1_ *b*_2_] = [*a*_1_ *a*_2_ *b*_1_ *b*_2_]
EL	entity linking model
BERT_BASE_	the pretrained BERT model

Algorithm 1 is used for training of TransE embedding:

Algorithm 2 is used for training of TransEA embedding:

Algorithm 3 is used for training of Complex embedding:

Algorithms 4 and 5 are used for training of the scoring model and the score alignment model, respectively:

Algorithm 6 is used for deriving implicit relations:

Algorithms 7, 8, and 9 are used for fine-tuning of the plain EL entity–question biencoder on BERT, the joint EL and ER entity–question biencoder on BERT, and SMILES name recognition on BERT, respectively:

Algorithm 10 is used for training of TransRA embedding:

## Nomenclature

AM: assembly model
BERT: bidirectional encoder representations from transformers
CBU: chemical building unit
CGQA: cross graph question answering
CID: compound identifier
DFT: density functional theory
dKG: dynamic knowledge graph
DL: description logic
EL: entity linking
ER: entity recognition
GBU: generic building unit
IRI: internationalized resource identifier
IR: information retrieval
IUPAC: International Union of Pure and Applied Chemistry
JPS: J-Park Simulator
LDF: linked data fragment
LLM: large language model
MOP: metal organic polyhedra
NER: named entity recognition
NLP: natural language processing
PCE: power conversion efficiency
PLM: pretrained language model
SMILES: simplified molecular-input line-entry system
SPARQL: SPARQL protocol and RDF query language
SP: semantic parsing
TWA: the world avatar
